# Enalapril Diminishes the Diabetes-Induced Changes in Intestinal Morphology, Intestinal RAS and Blood SCFA Concentration in Rats

**DOI:** 10.3390/ijms23116060

**Published:** 2022-05-27

**Authors:** Kinga Jaworska, Wojciech Kopacz, Mateusz Koper, Mateusz Szudzik, Marta Gawryś-Kopczyńska, Marek Konop, Tomasz Hutsch, Dawid Chabowski, Marcin Ufnal

**Affiliations:** Laboratory of Centre for Preclinical Research, Department of Experimental Physiology and Pathophysiology, Medical University of Warsaw, Pawinskiego 3c Street, 02-106 Warsaw, Poland; w.kopacz@icloud.com (W.K.); mateuszkoper988@gmail.com (M.K.); mateusz.szudzik@wum.edu.pl (M.S.); mgawrys1@wum.edu.pl (M.G.-K.); marek.konop@wum.edu.pl (M.K.); t.hutsch@yahoo.com (T.H.); chabowskidawid@gmail.com (D.C.); mufnal@wum.edu.pl (M.U.)

**Keywords:** intestinal RAS, diabetes, ACE-I, SCFA, TMAO

## Abstract

Evidence suggests that microbiota-derived metabolites, including short-chain fatty acids (SCFAs) and trimethylamine-oxide (TMAO), affect the course of diabetic multiorgan pathology. We hypothesized that diabetes activates the intestinal renin–angiotensin system (RAS), contributing to gut pathology. Twelve-week-old male rats were divided into three groups: controls, diabetic (streptozotocin-induced) and diabetic treated with enalapril. Histological examination and RT-qPCR were performed to evaluate morphology and RAS expression in the jejunum and the colon. SCFA and TMAO concentrations in stools, portal and systemic blood were evaluated. In comparison to the controls, the diabetic rats showed hyperplastic changes in jejunal and colonic mucosa, increased plasma SCFA, and slightly increased plasma TMAO. The size of the changes was smaller in enalapril-treated rats. Diabetic rats had a lower expression of Mas receptor (MasR) and angiotensinogen in the jejunum whereas, in the colon, the expression of MasR and renin was greater in diabetic rats. Enalapril-treated rats had a lower expression of MasR in the colon. The expression of AT1a, AT1b, and AT2 receptors was similar between groups. In conclusion, diabetes produces morphological changes in the intestines, increases plasma SCFA, and alters the expression of renin and MasR. These alterations were reduced in enalapril-treated rats. Future studies need to evaluate the clinical significance of intestinal pathology in diabetes.

## 1. Introduction

Intestinal digestion, absorption and hormonal activity are of major importance for the normal functioning of the whole organism. Numerous homeostatic processes in the intestines, such as water–electrolyte balance, digestion, secretion, and peptide absorption, are regulated by the intestinal renin–angiotensin system (RAS). The hyperactivity of the local RAS in the retina or kidneys may contribute to diabetic complications [1,2,3], but data on the intestinal RAS in diabetes are scant.

The intestines, especially the colon, constitute a major site for microbiome–host interactions. Ample evidence points to the pleiotropic effects of gut bacteria on the host [4,5]. For example, gut microbiota produces biologically active compounds that affect metabolic homeostasis [6]. Recently, bacteria-derived trimethylamine N-oxide (TMAO) has been associated with diabetes risk [7,8]. Short-chain fatty acids (SCFAs), as intestinal bacteria-derived metabolites, appear to have beneficial effects on metabolism [9].

Furthermore, there is evidence that bacterial metabolites may affect the functioning of the RAS. For example, SCFAs modulate blood pressure via the local RAS in the kidneys [10] and TMAO treatment alters the expression of RAS receptors in the heart [11].

We hypothesized that diabetes activates the intestinal RAS which contributes to gut pathology and increases the blood concentration of microbiota-derived metabolites. Therefore, in this study, we assessed the intestinal barrier morphology, intestinal RAS gene expression and concentration of TMAO and SCFAs in the portal and systemic circulation in a rat model of diabetes.

## 2. Results

The study was conducted on three experimental groups: (i) a control (sham) group (*n* = 12), (ii) a diabetic (streptozotocin-induced) group (*n* = 17), and (iii) an ACE-I group (diabetic rats receiving enalapril, an angiotensin-converting enzyme inhibitor, for 4 weeks, *n* = 14). Tissues, blood and stool samples were obtained 4 weeks after streptozotocin or saline (control) administration. Histological examination and RT-qPCR were performed to evaluate morphology and RAS expression in the jejunum and colon. The concentrations in the stool, portal and systemic blood of TMAO and the following SCFAs were evaluated: acetic acid (AA), propionic acid (PA), isobutyric acid (IBA), butyric acid (BA), 2-methylbutyric acid (2MeB), isovaleric acid (IVA), valeric acid (VA), isocaproic acid (ICA), and caproic acid (CA).

### 2.1. Diabetes Produces Morphological Changes in the Intestines

**Figure 1:**
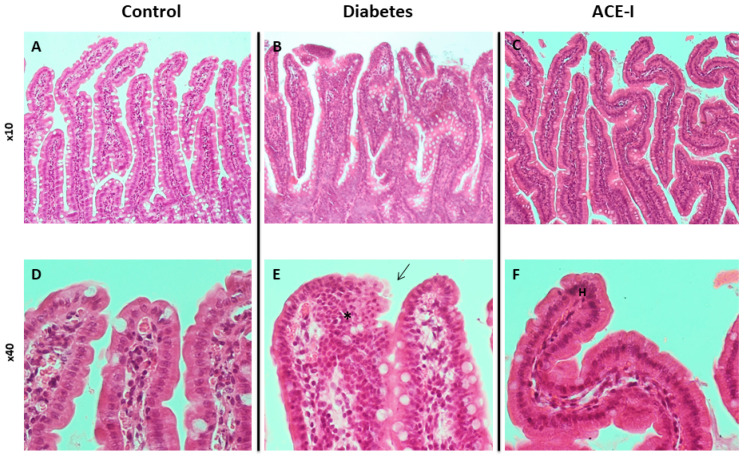
Histopathology of the jejunum in rats of the control (*n* = 7), diabetes (*n* = 7) and ACE-I (*n* = 7) groups. The diabetes group was assessed 4 weeks after streptozotocin administration; the ACE-I group comprised diabetic rats treated with enalapril for 4 weeks. (**A**) Mucosa of the jejunum in the control (×10); (**B**) mucosa of the jejunum in rats with diabetes (×10); (**C**) mucosa of the jejunum in rats with diabetes treated with enalapril (×10); (**D**) villi of jejunum mucosa in the control (×40); (**E**) villi of jejunum mucosa in diabetes (×40), disturbed and discontinuous structure of the brush border (arrow), dysplastic changes (asterisk); (**F**) villi od jejunum mucosa in the ACE-I group (×40); hyperplasia (H).

**Figure 2:**
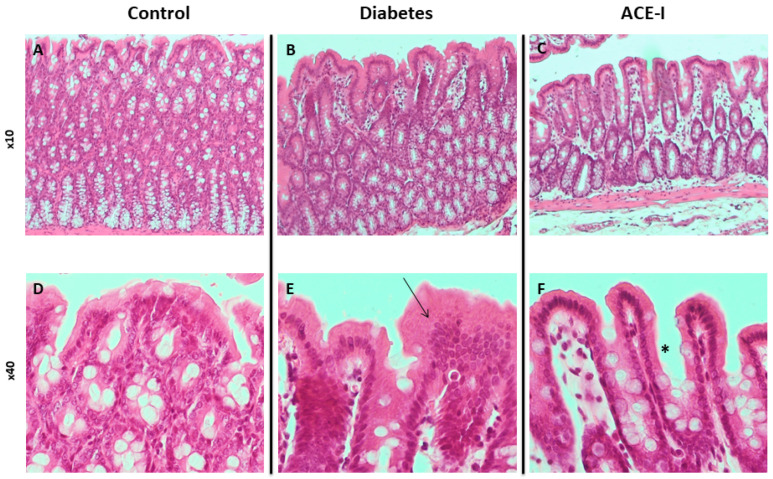
Histopathology of the colon in rats of the control (*n* = 7), diabetes (*n* = 7) and ACE-I (*n* = 7) groups. Diabetes group were assessed 4 weeks after streptozotocin administration and the ACE-I group comprised diabetic rats treated with enalapril for 4 weeks. (**A**) Colonic mucosa in the control (×10); (**B**) colonic mucosa in rats with diabetes (×10); (**C**) colonic mucosa in rats with diabetes treated with enalapril (×10); (**D**) colonic mucosa in the control (×40); (**E**) colonic mucosa in diabetes (×40) presenting with an increase in height and cellularity (arrow); (**F**) colonic mucosa in the ACE-I group (×40) presenting with an increase in height but normal cellularity (asterisk).

The control group had no signs of pathological changes in the jejunum or colon (Figure 1A,D and Figure 2A,D).

The diabetic group revealed pronounced morphological changes in the jejunum, including hyperplasia of the mucous membrane (hyperplasia of enterocytes, double-row enterocyte testicular system), dysplastic changes in the intestinal epithelium, disturbed and discontinuous structure of the brush border, increased mucus on the surface of the mucosa, and an increase in stromal cellularity caused by connective tissue hyperplasia. Hyperplastic changes were confirmed in the morphometric analysis; the diabetic group showed increased crypt depth and villus height in comparison to the control group (*p* = 0.025 and *p* = 0.006, respectively). Additionally, the diabetic group showed increased lymphocytic stromal infiltration, slight features of edema, and degenerative changes of nerve ganglion cells in the submucosa and muscle membrane arterioles with features of endothelial stimulation and the vacuolation of tunica intima. Colons of the diabetic group showed an increase in height and cellularity of the colonic mucosa (Figure 1B,E and Figure 2B,E). Morphometric analysis did not show significant differences in the number of goblet cells (per 50 enterocytes) and number of mitoses in crypts between the groups. Detailed results of the morphometric analysis are shown in the Supplementary Materials (Supplementary Results, Supplementary Figures S1 and S2).

Diabetic rats receiving enalapril (ACE-I group) showed smaller hyperplastic and dysplastic changes in the jejunal mucosa than the diabetic group. Similarly, hyperplastic changes and the infiltration of lymphocytes in the stroma were less pronounced than in the diabetic group. Colons of the ACE-I group showed an increased height of colonic mucosa (Figure 1C,F and Figure 2C,F).

### 2.2. Diabetes Alters the mRNA Expression of Intestinal RAS

**Figure 3:**
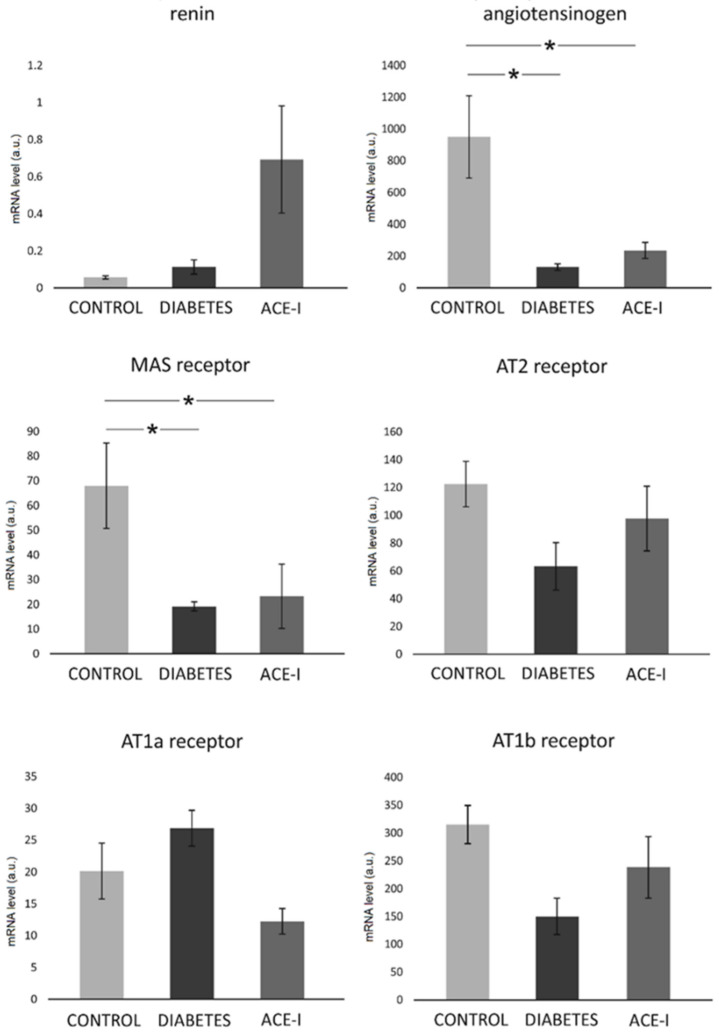
The expression of renin–angiotensin system components in the jejunum. Tissues were obtained from diabetic rats on water 4 weeks after streptozotocin administration (DIABETES; *n* = 5); diabetic rats treated with enalapril for 4 weeks (ACE-I, *n* = 5); and healthy rats (CONTROL, *n* = 5). AT1a receptor—angiotensin II receptor type 1a; AT1b receptor—angiotensin II receptor type 1b; AT2 receptor—angiotensin II receptor type 2. Values are means ± SE; *—*p* < 0.05 by one-way ANOVA followed by Duncan’s test.

In the jejunum, there were significant differences between the groups with respect to the Mas receptor (F2,12 = 3.89, *p* = 0.049) and angiotensinogen (F2,12 = 7.11, *p* = 0.009). Namely, the diabetic rats on water and enalapril (ACE-I group) showed significantly decreased mRNA expression compared to the healthy control rats. There were no significant changes in the jejunal expression of renin, or the AT1a, AT1b, or AT2 receptors, as shown in Figure 3.

**Figure 4:**
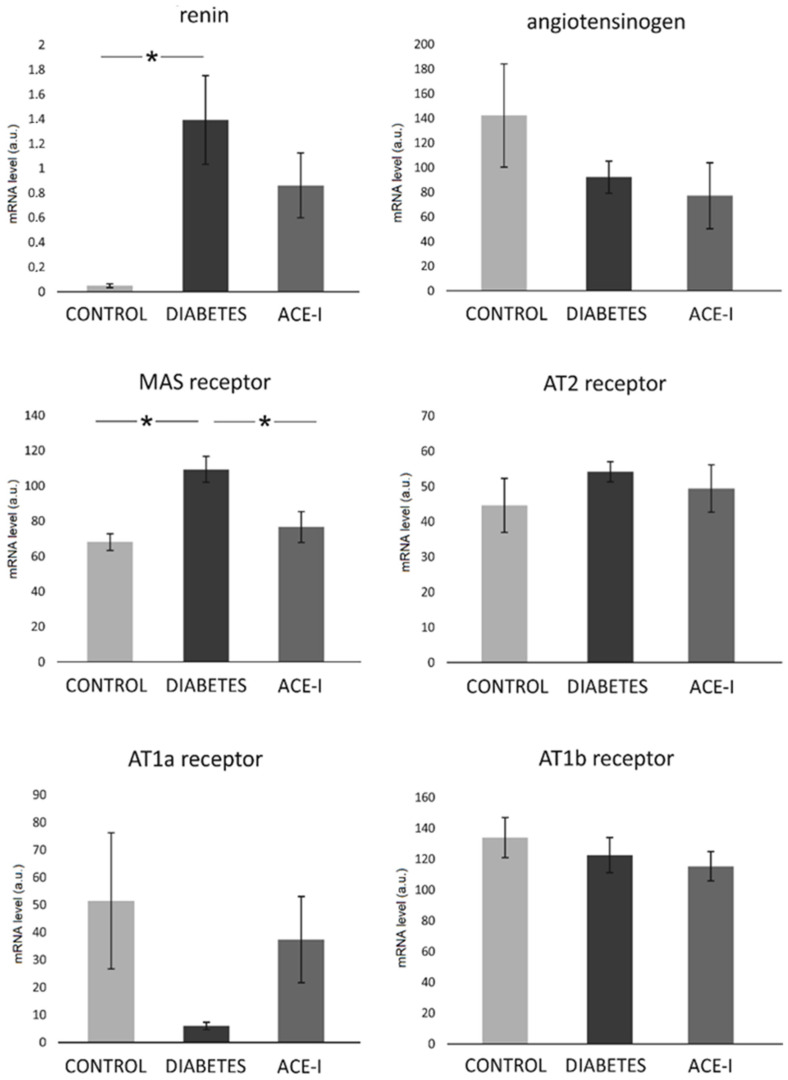
The expression of renin–angiotensin system components in the colon. Tissues were obtained from diabetic rats on water 4 weeks after streptozotocin administration (DIABETES; *n* = 5); diabetic rats treated with enalapril for 4 weeks (ACE-I, *n* = 5); and healthy rats (CONTROL; *n* = 5). AT1a receptor—angiotensin II receptor type 1a; AT1b receptor—angiotensin II receptor type 1b; AT2 receptor—angiotensin II receptor type 2. Values are means ± SE; *—*p* < 0.05 by one-way ANOVA followed by Duncan’s test.

In the colon, significant differences were observed in the mRNA expression of the Mas receptor (F2,12 = 7.68, *p* = 0.007) and renin (F2,12 = 4.66, *p* = 0.03). Diabetic rats demonstrated a higher expression of the Mas receptor and renin, as shown in Figure 4. The ACE-I group showed lower Mas expression than the diabetic group. There were no significant differences between the groups in terms of the colonic expression of angiotensinogen, or the AT1a, AT1b, or AT2 receptors, as shown in Figure 4.

### 2.3. Diabetes Increases the Portal and Systemic Concentration of Bacterial Metabolites

#### 2.3.1. Concentration in the Portal Vein

**Figure 5:**
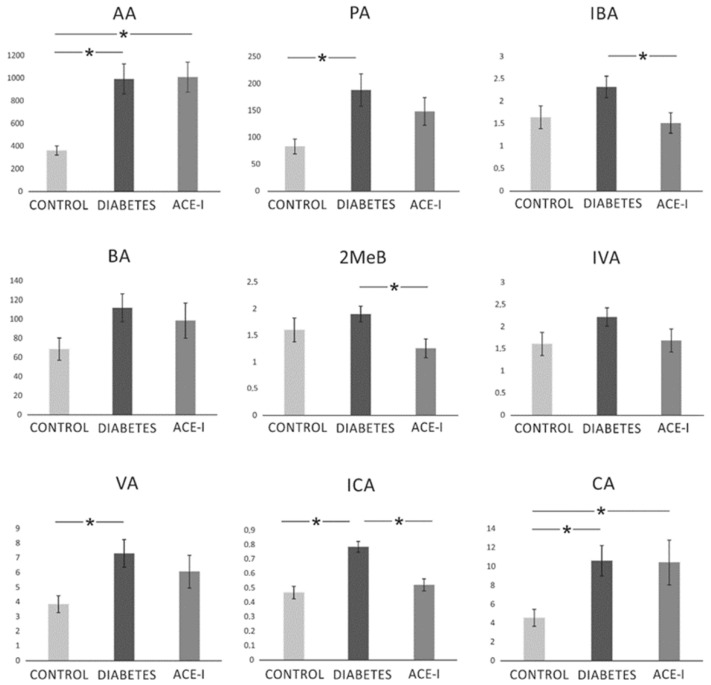
The portal concentration of short-chain fatty acids. Portal vein blood samples were obtained from diabetic rats on water 4 weeks after streptozotocin administration (DIABETES; *n* = 15); diabetic rats treated with enalapril for 4 weeks (ACE-I; *n* = 12); and healthy rats (CONTROL; *n* = 12). AA—acetic acid; PA—propionic acid; IBA—isobutyric acid; BA—butyric acid; 2MeB—2-methylbutyric acid; IVA—isovaleric acid; VA—valeric acid; ICA—isocaproic acid/4-methylvaleric acid; CA—caproic acid. Values are means ± SE; *—*p* < 0.05 by one-way ANOVA followed by Duncan’s test.

There were significant differences between the groups in the portal concentration of the following SCFAs: AA (F2,35 = 9.07, *p* < 0.001), PA (F2,35 = 4.14, *p* = 0.024), IBA (F2,35 = 3.46, *p* = 0.043), MeB (F2,35 = 3.40, *p* = 0.044), VA (F2,35 = 3.49, *p* = 0.042), ICA (F2,35 = 18.40, *p* < 0.001), and CA (F2,35 = 3.55, *p* = 0.039). In general, the diabetic group demonstrated an increase in SCFA portal concentration. In comparison to the diabetic group, the ACE-I group showed lower concentrations of IBA, 2MeB, and ICA. Detailed results are presented in Figure 5.

#### 2.3.2. Concentration in the Systemic Venous Blood

**Figure 6:**
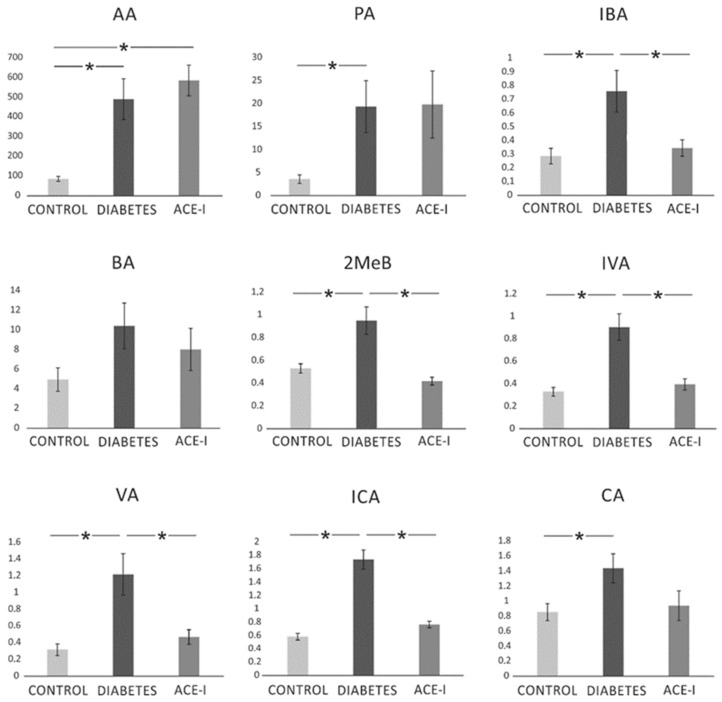
The systemic concentration of short-chain fatty acids. Systemic blood samples were obtained from diabetic rats on water 4 weeks after streptozotocin administration (DIABETES; *n* = 15); diabetic rats treated with enalapril for 4 weeks (ACE-I; *n* = 12); and healthy rats (CONTROL; *n* = 12). AA—acetic acid; PA—propionic acid; IBA—isobutyric acid; BA—butyric acid; 2MeB—2-methylbutyric acid; IVA—isovaleric acid; VA—valeric acid; ICA—isocaproic acid/4-methylvaleric acid; CA—caproic acid. Values are means ± SE; *—*p* < 0.05 by one-way ANOVA followed by Duncan’s test.

There were significant differences between the groups in the systemic concentration of the following SCFAs: AA (F2,35 = 10.77, *p* < 0.001), PA (F2,34 = 4.06, *p* = 0.026), IBA (F2,33 = 5.76, *p* = 0.007), MeB (F2,34 = 11.64, *p* < 0.001), IVA (F2,34 = 14.70, *p* < 0.001), VA (F2,34 = 8.33, *p* = 0.001), ICA (F2,34 = 39.98, *p* < 0.001), and CA (F2,34 = 3.48, *p* = 0.042). In general, diabetes induced an increase in SCFA portal concentration. ACE-I group showed lower concentrations of IBA, 2MeB, IVA, VA, and ICA compared to the diabetic group. Detailed results are presented in Figure 6.

Additionally, we investigated the plasma concentration of TMAO in the systemic circulation. Diabetic rats tended to have higher TMAO concentrations than the control or ACE-I group. However, the differences between the groups did not reach significance (*p* = 0.08; data not shown).

#### 2.3.3. Concentration in Stools

Table 1 presents the fecal concentrations of the SCFAs. The diabetic and ACE-I groups had a higher AA concentration in the stool samples than the controls. There were no differences in fecal concentration of the other evaluated SCFAs.

## 3. Discussion

The novel findings of our study are that diabetes disturbs colonic morphology, changes the expression of several RAS proteins in the small intestine and colon, and increases SCFA portal and systemic concentrations. Furthermore, we found that enalapril treatment may partly reverse reported diabetes-induced changes.

Diabetes is a significant medical and social problem, reducing life quality and causing multiple organ complications and premature mortality. Importantly, there is evidence that diabetes disturbs intestinal homeostasis [12,13].

The intestine is an essential homeostatic organ responsible for nutrient absorption and interaction with abundant gut microbiomes. Some key intestinal processes are regulated by the intestinal RAS, which plays a role in digestion, peptide transport, and water–electrolyte homeostasis [14]. The intestinal RAS is also a major regulator of glucose uptake in the intestines [15].

A growing body of evidence demonstrates that gut bacteria-derived metabolites exert beneficial and harmful effects on metabolism, the circulatory system, and the RAS [6,11,14]. For example, circulating TMAO has been associated with increased diabetes risk [7,8]. On the other hand, SCFAs have been reported to regulate host energy homeostasis positively. For example, SCFA inhibited the fat accumulation in the adipose tissue by the suppression of adipose insulin signaling [16]. Additionally, acetate and butyrate have been shown to protect against type 1 diabetes by immunomodulating action [17].

The histological assessment in our study showed that diabetes produces pronounced modifications to the morphology of the small intestine and colon, including the hyperplasia of the mucous membrane and dysplastic changes in the intestinal epithelium. Our results support previous reports demonstrating diabetes-induced morphological disturbances in the small intestine [18].

Changes in the plasma concentrations of bacteria-derived metabolites accompany alterations in the gut structure. Diabetes increased the portal and systemic levels of SCFAs and tended to increase TMAO as well. Since the concentrations of the SCFAs in the stool samples were virtually unaffected, it can be speculated that their increase in plasma concentration may be a consequence of intestinal barrier disruption. In fact, diabetes is known to produce higher gut barrier permeability to sugars [12]. Bacterial metabolites are an important element in the interaction of the gut microbiome with the host organism. Recent studies have suggested that gut bacteria play an essential role in the pathophysiology of diabetes [19]. However, it should be pointed out that alterations in intestinal morphology and permeability caused by diabetes may shape the microbial habitat and affect the availability and systemic effects of bacterial products.

Our study showed several changes in the mRNA expression of the intestinal RAS components of diabetic rats. We did not observe any significant changes in the expression of the AT1a or AT1b receptors which are typically related to the adverse effects of exaggerated RAS activation. In the jejunum, diabetes decreased the expression of the Mas receptors and angiotensinogen mRNA levels. Mas receptors are a major component of the ACE2-MAS axis, which is beneficial and protective for the intestinal mucosa and glucose metabolism [14]. Therefore, decreased Mas receptor expression in the jejunum may reflect the pathological changes caused by diabetes. Our results contradict those obtained by Wong et al., who demonstrated the upregulation of angiotensinogen and Mas receptors in the jejunum of diabetic rats [20,21]. However, the Wong group assessed changes 2 weeks after the induction of diabetes while, in our study, data were collected after 4 weeks. We speculate that the increase in ACE2-MAS components is a compensatory response that recedes over time.

To the best of our knowledge, this is the first study showing diabetic changes in the colonic RAS. Conversely to the jejunum, we observed an increase in the renin and Mas receptor expression in the diabetic group. It may be hypothesized that these location-dependent differences are associated with abundant microbiomes in the colon. Available data suggests that probiotics increase the hepatic expression of the Mas receptor [22]. However, the evidence of the direct action of the microbiome on colonic Mas is yet to be discovered.

Finally, the present study demonstrated that enalapril ameliorated pathological changes in gut morphology. In this regard, the blockade of the local RAS provides beneficial effects in diabetic nephropathy [1,23] and retinopathy [2,3]. For instance, angiotensin II antagonists have been shown to reduce albuminuria [24] and slow the progression of renal disease from microalbuminuria to macroalbuminuria [25]. With respect to gastrointestinal function, ACE inhibition or angiotensin receptor blockades have been reported to counteract intestinal inflammation [26,27], colorectal cancer [28], and hypertension-induced intestinal damage [29] in rodent models. A limitation of our study is that we did not include the group receiving enalapril without diabetes induction. This would give valuable information regarding the influence of enalapril on the healthy intestine. However, the purpose of our study was to assess enalapril usefulness in the context of diabetes treatment. Currently, there are limited data about the effect of such therapy on the intestinal impairments associated with diabetes.

## 4. Materials and Methods

### 4.1. Ethical Approval

The animal experiments were carried out according to the Directive 2010/63/EU and were approved by the Local Bioethical Committee (616/2018).

The study was conducted on 3 experimental groups: (i) a control (sham) group (*n* = 12), (ii) a diabetic group (*n* = 17) and (iii) an ACE-I group (diabetic rats receiving enalapril, *n* = 14).

Induction of diabetes in rats

Male, 12-week-old Sprague Dawley rats were fasted overnight before the procedure. Diabetic and ACE-I groups received streptozotocin (STZ, Merck, Warsaw, Poland) at a dose of 65 mg/kg of body weight (BW) dissolved in 0.01 mol/L citrate buffer (pH 4.5) in a single intraperitoneal injection. Afterward, rats were placed in cages with food and water ad libitum. Fasting glucose levels above 250 mg/dL at 3 and 7 days post-STZ injection was considered a successful diabetes induction. Rats with ineffective diabetes induction were not included in the analysis. Eventually, 15 rats from the diabetic group, 12 rats from the ACE-I group and 12 rats from the control group were included in the analysis. The ACE-I group was treated with enalapril (Polpharma, Warsaw, Poland) dissolved in 50 mg/L drinking water (a dose of 6.5 ± 0.3 mg/kg BW/d) for 4 weeks. We decided to use a non-antihypertensive dose (based on our previous studies and the literature (Jaworska 2017, Piotrkowski 2009)) to evaluate effects independent of hemodynamic changes. The diabetic and control groups were maintained on tap water.

### 4.2. Tissue, Blood, and Fecal Sample Collection and Processing

Four weeks after the induction of diabetes, rats were anesthetized with urethane (Sigma-Aldrich, Kraków, Poland) at a dose of 1.5 g/kg BW. The portal vein and femoral vein were catheterized as previously described [30]. Chilled EDTA tubes were used to collect blood samples and were centrifuged for 5 min at 5000 rpm at 4 °C. The obtained plasma was collected into Eppendorf tubes and frozen at −20 °C. After blood collection, the rats were euthanized by decapitation, and stool samples (0.5 mL) from the middle part of the colon were collected. The jejunum and colon were harvested. Tissues sections for molecular biology studies were immediately frozen at 80 °C. Tissues dedicated for histological analysis were fixed in 10% buffered formalin. Stool samples were weighted and homogenized with 1 mL of 0.9% NaCl by vortexing them for 5 min. The samples were then centrifuged for 12 min at 8000 rpm and supernatants were transferred into a laboratory tube and centrifuged again for 12 min. Procedures were performed at 2–5 °C. The supernatants were collected into the Eppendorf tubes and frozen at −20 °C.

### 4.3. Renin–Angiotensin System Analysis by RT-qPCR

Mas receptor, renin, angiotensinogen, angiotensin receptor type 1a (AT1a receptor), angiotensin receptor type 1b (AT1b receptor), and angiotensin receptor type 2 (AT2 receptor) expression in the jejunum and colon samples were evaluated.

Total cellular RNA was extracted from approx. 15 mg of wet tissue of the jejunum and colon with Trizol reagent (Invitrogen, Carlsbad, CA, USA) according to the manufacturer’s protocols.

Next, using an Iscript^®^ (Bio-Rad, Hercules, CA, USA), 1.5 µg of the total DNAse-treated RNA was reverse-transcribed. Real-time quantitative PCR analysis was performed via a Bio-Rad real-time system using gene-specific primer pairs (see Table S1 in the Supplemental Material). The amplified products were detected with iTaq^®^ Universal SYBR Green Supermix (Bio-rad, Hercules, CA, USA). Melting curve analysis and agarose gel electrophoresis of the PCR products confirmed amplification specificity. Data analysis was performed using Bio-Rad CFX Maestro Software (version 0.953, MOMA, Aarhus, Denmark). Transcript levels were normalized relative to the reference genes: HPRT for the jejunum and β-actin for the colon were selected from 4 different housekeeping genes using the NormFinder software.

### 4.4. Histological Assessment

Tissue sections were dehydrated using graded ethanol and xylene baths and embedded in paraffin wax. Sections of 3–4 µm were stained with hematoxylin and eosin (HE). General histological examination was performed at the magnification of 10×, 40× and 100× (objective lens) and 10× (eyepiece) and photographic documentation was made. The mucosa and submucosa of the colon and jejunum, intestinal crypts and their cell composition, mucosa and submucosa blood vessels were assessed. Morphometric measurements were performed at a magnification of 40× (objective lens).

### 4.5. Evaluation of Bacterial Metabolite Concentration

Intestinal permeability was estimated by the direct measurement of bacterial metabolite concentration in the portal vein and the analysis of stool concentration.

Plasma and stool concentrations of the following bacterial metabolites were measured: TMAO and SCFAs: AA—acetic acid (C2); PA—propionic acid (C3); IBA—isobutyric acid (C4); BA—butyric acid (C4); 2MeB—2-methylbutyric acid (C5); IVA—isovaleric acid (C5); VA—valeric acid (C5); ICA—isocaproic acid/4-methylvaleric acid (C6); and CA—caproic acid (C6). Concentrations were evaluated using liquid chromatography coupled with triple-quadrupole mass spectrometry. The instrumentation consisted of the Waters Acquity Ultra-Performance Liquid Chromatograph coupled with the Waters TQ-S triple-quadrupole mass spectrometer. The mass spectrometer operated in the multiple-reaction monitoring (MRM)-positive electrospray ionization (ESI) mode, as described in detail previously [30].

### 4.6. Data Analysis and Statistics

Blinding was provided for data analysis. The Kolmogorov–Smirnov test was used to test the normality of the distribution. The differences between the groups were evaluated by one-way ANOVA, followed by Duncan’s new multiple range test (MRT). A value of two-sided *p* < 0.05 was considered significant. Quantitative data are expressed as mean and standard error (SE). Analyses were conducted using Dell Statistica, version 13 (Dell Inc., Tulsa, OK, USA).

## 5. Conclusions

Diabetes produces pronounced morphological changes in the gut, increasing the penetration of bacterial metabolites to circulation. Our study shows that these changes are accompanied with alterations in local small intestinal and colonic RAS; however, it needs to be elucidated whether these observations are related to the presence of gut microbiota and their metabolites. Since diabetes-induced changes in the intestine may be partly reversed by enalapril, intestines may be a therapeutic target for ACE inhibitors. However, more studies are needed to better understand the significance of intestinal pathology in diabetes and the relevance of RAS blockades in diabetic intestines.

## Figures and Tables

**Table 1:** Fecal concentrations of short-chain fatty acids (SCFAs).

SCFA	Control Group (µmol/L)	Diabetic Group (µmol/L)	ACE-I Group (µmol/L)
acetic acid (AA)	50,425 ± 2904.1	64,221 ± 3742.9 *	71,865 ± 4991.3 *
propionic acid (PA)	13,758 ± 768.5	15,931 ± 1078.6	17,740 ± 1619.3
isobutyric acid (IBA)	398 ± 41.4	377 ± 28.9	336 ± 44.5
butyric acid (BA)	10,781 ± 1047.2	13,978 ± 1618.1	16,440 ± 2019.2
2-methylbutyric acid (2MeB)	226 ± 31.1	246 ± 21.2	205 ± 29.7
isovaleric acid (IVA)	325 ± 37.5	335 ± 26.3	330 ± 46.2
valeric acid (VA)	728 ± 80.7	906 ± 86.8	972 ± 147.3
isocaproic acid (ICA)	15 ± 4.4	18 ± 2.8	21 ± 5.2
caproic acid (CA)	467 ± 90.3	1003 ± 223.8	1339 ± 359.5

Diabetic rats on water 4 weeks after streptozotocin administration (DIABETES; *n* = 15); diabetic rats treated with enalapril for 4 weeks (ACE-I; *n* = 12); and healthy rats (CONTROL; *n* = 12). Values are means ± SE; *—*p* < 0.05 vs. control group by one-way ANOVA followed by Duncan’s test.

## Data Availability

Not applicable.

## Supplementary Materials

The following supporting information can be downloaded at: https://www.mdpi.com/article/10.3390/ijms23116060/s1.
