# Supplementary material for: Enalapril Diminishes the Diabetes-Induced Changes in Intestinal Morphology, Intestinal RAS and Blood SCFA Concentration in Rats
Source: Int J Mol Sci. 2022 May 27;23(11):6060. doi: 10.3390/ijms23116060 (PMC9181110; doi:10.3390/ijms23116060)
Supplement: Supplementary file 1 [file ijms-23-06060-s001.zip › ijms-1725094-supplementary.pdf]

SUPPLEMENTARY MATERIALS

Supplementary Results

Histological morphometric analysis of the jejunum and colon

In the jejunum, there were significant differences between the groups with respect to crypt depth ($F_{2,18}$ =4.44, p=0.027) and height of the villi ($F_{2,18}$ =7.50, p=0.004). Namely, diabetic group had increased crypt depth in comparison to control group and diabetic rats on water and enalapril (ACE-I group) showed significantly increased height of the villi compared to healthy control rats. There were no significant changes in the height of the enterocyte, the number of goblet cells (per 50 enterocytes), and the number of mitoses in crypts, Supplementary Figure 1.

In the colon, morphometric analysis revealed no significant differences in crypt depth, the height of the enterocyte, the number of goblet cells (per 50 enterocytes), and the number of mitoses in crypts between the groups, Supplementary Figure 2.

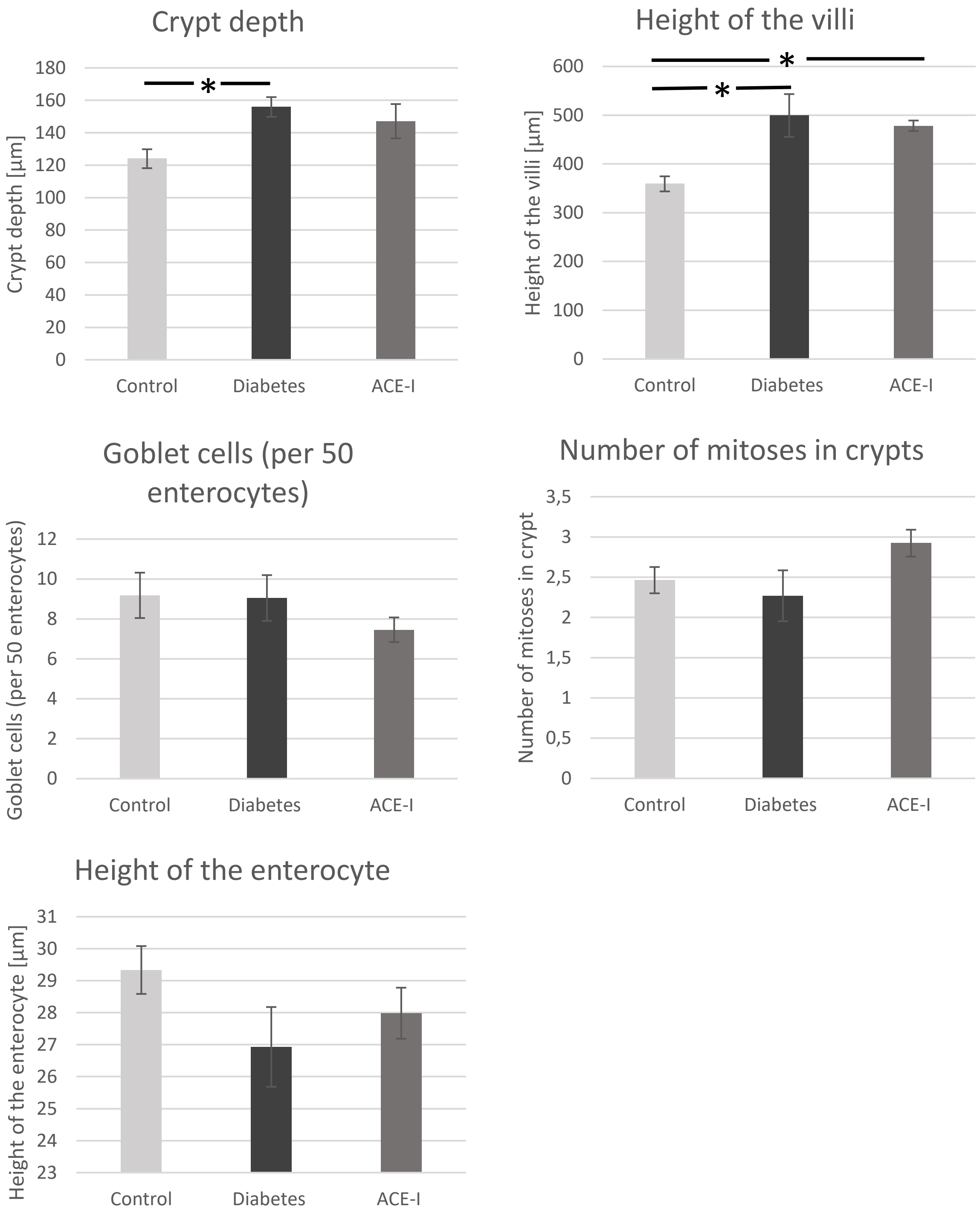

Figure S1. Histological morphometric analysis of the jejunum in rats of the control (n=7), diabetes (n=7) and ACE-I (n=7) groups. Diabetes group have been assessed 4 weeks after streptozotocin administration, ACE-I group are diabetic rats treated with enalapril for 4 weeks. Values are means ± SE, * - $p<0.05$, by one-way ANOVA followed by Tukey's test.

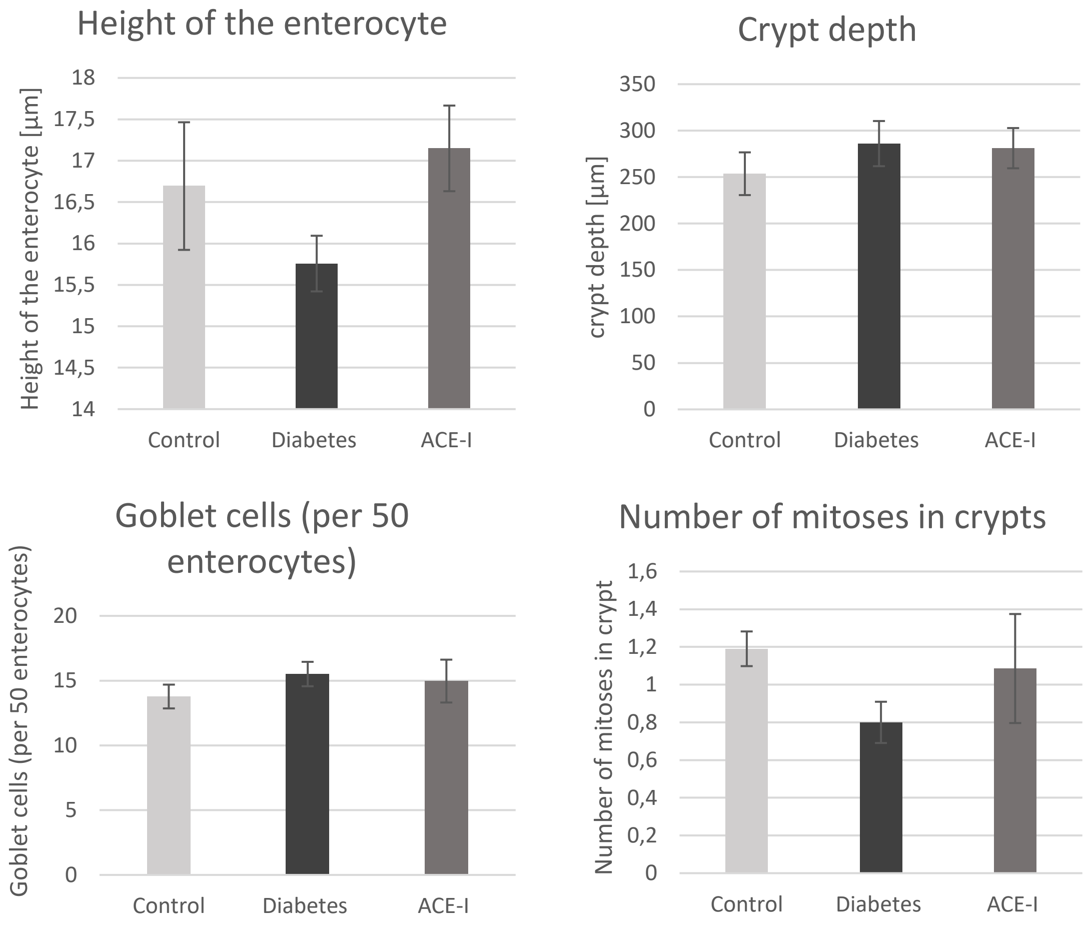

Figure S2. Histological morphometric analysis of the colon in rats of the control (n=7), diabetes (n=7) and ACE-I (n=7) groups. Diabetes group have been assessed 4 weeks after streptozotocin administration, ACE-I group are diabetic rats treated with enalapril for 4 weeks. Values are means ± SE, * - $p<0.05$, by one-way ANOVA followed by Tukey's test.

Table S1. List of oligonucleotide primers used for RT-qPCR

Gene	Encoded product	Unique Biorad Assay ID	PCR product size [bp]	Ensembl Accession number
Hprt1	Hypoxanthine-guanine phosphoribosyltransferase	qRnoCED0057020	79	ENSRNOG00000031367
Actb	Beta-Actin	qRnoCED0018219 q	95	ENSRNOG00000043292
Ren	Renin	qRnoCID0008721	115	ENSRNOG00000002937
Agt	Angiotensinogen	qRnoCED0051666	95	ENSRNOG00000018445
Agtr1a	Type-1A angiotensin II receptor	qRnoCID0052626	68	ENSRNOG00000018346
Agtr1b	Type-1B angiotensin II receptor	qRnoCED0005729	90	ENSRNOG00000010640
Agtr2	Type-2 angiotensin II receptor	qRnoCED0007551	83	ENSRNOG00000050006
Gapdh	glyceraldehyde-3-phosphate dehydrogenase	qRnoCID0057018	115	ENSRNOG00000018630
Mas1	Proto-oncogene Mas	qRnoCED0009257	114	ENSRNOG00000014971
